# Dyslipidaemia and cardiovascular risk – Key considerations in South Asians

**DOI:** 10.1016/j.clinme.2026.100571

**Published:** 2026-04-07

**Authors:** Aneesha Chauhan, Shaan Sahota, Lavandan Jegatheeswaran, Haneesh Kaur Johal, Rubin Minhas, Prashanth Patel, Pankaj Gupta, Vinod Patel, Amitava Banerjee, Kiran Patel, Anvesha Singh

**Affiliations:** a King’s College Hospital NHS Foundation Trust, London, UK; b Bart’s Health NHS Trust, London, UK; c University Hospitals Coventry and Warwickshire, UK; d Birmingham Women's and Children's NHS Foundation Trust, Birmingham, UK; e Oakfield Health Centre, Gravesend, Kent, UK; f University Hospitals of Leicester NHS Trust, Leicester, UK; g NIHR Leicester Biomedical Research Centre, Leicester, UK; h British Heart Foundation Leicester Centre of Research Excellence, University of Leicester, Leicester, UK; i George Eliot Hospital NHS Trust, Nuneaton, UK; j Warwick Medical School, University of Warwick, Coventry, UK; k Institute of Health Informatics, University College London, London, UK; l University Hospitals Birmingham NHS Foundation Trust, Birmingham, UK

**Keywords:** Dyslipidaemia, Cardiovascular disease, Cardiovascular mortality and morbidity, South Asians, Lipid lowering therapy

## Abstract

Cardiovascular disease remains the leading global cause of mortality. It disproportionately affects South Asians, who face an approximately two-fold higher risk of atherosclerotic cardiovascular disease (CVD), with a particularly pronounced excess risk of coronary heart disease and myocardial infarction, when compared to White Caucasians. Dyslipidaemia is a major modifiable CVD risk factor, with South Asians being predisposed to more atherogenic lipid profiles. Understanding cardiovascular risk in South Asian populations requires recognising key differences in risk factors, disease presentation and treatment strategies compared with other ethnic groups. This narrative review highlights the need for targeted research and culturally tailored interventions to optimise dyslipidaemia management and reduce the disproportionate burden of CVD in South Asians.

## Graphical Abstract

**Figure:**
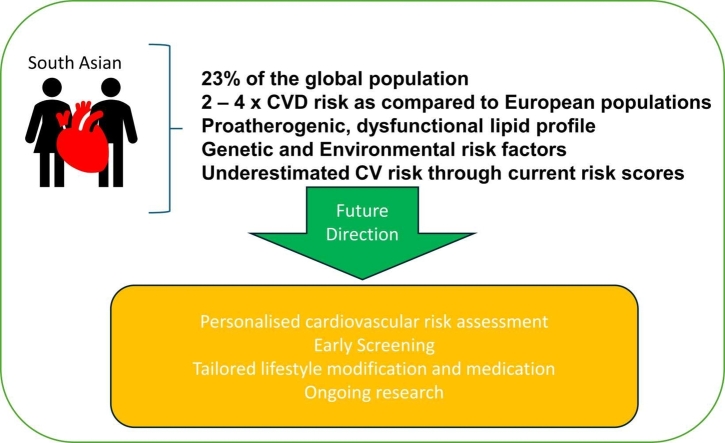

## Introduction

Cardiovascular disease (CVD) causes a quarter of all deaths in the UK and is the largest cause of mortality worldwide.^1^ Its impact extends beyond individuals to families, caregivers and economies.^2^ With the growing healthcare needs of an ageing, multimorbid population, CVD prevention is a research and policy priority.^2^ However, cardiovascular (CV) risk is not equally distributed. South Asians are 1.5 times more likely to die from coronary artery disease than White Caucasians.^3^ South Asian ancestry accounts for 1.8 billion individuals, or 23% of the global population^4^ – addressing population attributable risks is crucial. Dyslipidaemia is a major risk factor for CV events, with a cumulative risk over time.^5^ Given the higher prevalence of dyslipidaemia and more dysfunctional, atherogenic lipoproteins in South Asians, it is a key modifiable risk factor in this population. In this narrative review of dyslipidaemia in South Asians, we provide an overview of current evidence and practice and highlight areas for future research.

## Cardiovascular risk in people of South Asian heritage

There have been significant advances in CVD primary and secondary prevention over the last 50 years. In the UK, there has been a 30% reduction in incident atherosclerotic CVD over the last two decades, which has been attributed to improved risk factor modification.^6^ While this demonstrates the substantial benefit of preventative strategies, it is important to note differences in CVD rates by ethnicity remain.

South Asians are individuals with ethnic heritage from India, Pakistan, Bangladesh, Sri Lanka, Nepal, Bhutan and the Maldives. While this definition is a vast oversimplification of the genetic and environmental heterogeneity within this population, it has allowed researchers to highlight the disproportionate global burden of coronary artery disease (CAD) experienced by those of South Asian descent.^7^

Higher rates of CAD and CV mortality have been reported in South Asians since the 1940s.^8^ There is an estimated two- to four-fold higher risk of incident atherosclerotic CVD when compared with individuals of European ancestry.^4^, ^7^

**Fig. 1:**
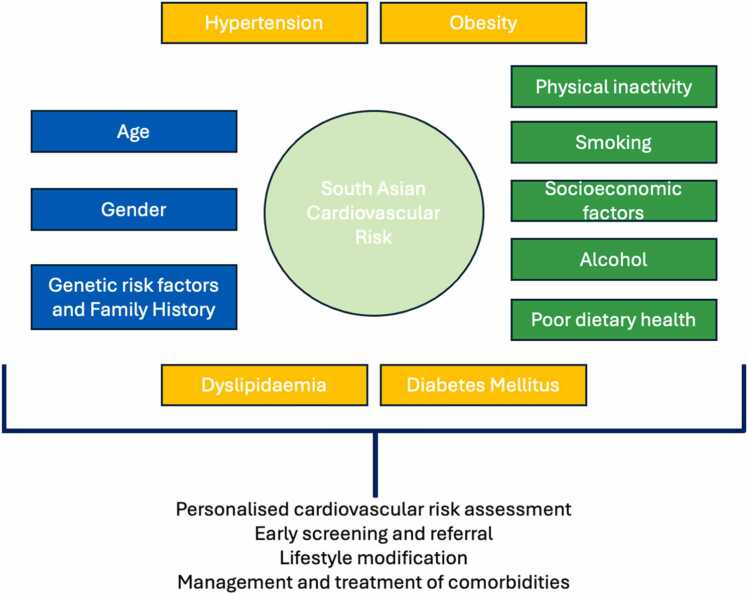
Known risk factors that increase cardiovascular risk in the South Asian population.^4^, ^8^ Blue = non-modifiable risk factors, Green = modifiable risk factors, yellow = modifiable risk factors that have an element of non-modifiable genetic risk.

The increased CV risk in South Asians is attributable to a combination of modifiable and non-modifiable factors (Fig. 1).^9^ In the landmark INTERHEART study, which examined modifiable risk factors associated with myocardial infarction (MI) within 52 countries, CV risk could be largely attributed to dyslipidaemia, smoking, diabetes, central obesity, hypertension, non-vegetarian diet, reduced physical activity, excess alcohol consumption and psychosocial factors.^7^, ^8^ However, there remains a residual CVD risk for South Asians once these modifiable risk factors are adjusted for, and genetics are thought to play a significant part.^4^, ^9^ The search for genetic and epigenetic loci specific to South Asian CVD risk is an area of growing research interest and offers the potential for future targeted therapeutic options. However, it must be acknowledged that the term ‘South Asian’ does not capture the vast genetic and environmental heterogeneity of its subpopulations, and there is variation in its definition between studies. Furthermore, there is relatively limited genetic analysis on South Asian cohorts, with incomplete recording of ethnicity creating further knowledge gaps – a challenge for those investigating genetic CVD risk.

## Ethnicity related differences in lipid patterns

Dyslipidaemia is a key contributor to the elevated CVD risk in South Asians. Similar to CVD risk as a whole, its prevalence is influenced by genetic and environmental factors, including socioeconomic status, diet, physical activity and age.^10^ Migration does not reduce this atherogenic burden, as South Asians in the USA and UK exhibit worse lipid profiles than Chinese and European populations.^8^ However, it is not simply the levels of various lipoproteins that are important when assessing risk, but the functionality, or dysfunctionality, of those proteins.

### Low-density lipoprotein cholesterol (LDL-C)

Of all the proatherogenic lipid markers, LDL-C is currently the principal marker on which clinical decisions are based. It is well correlated with CV mortality, hence its widespread use.^5^ However, it may not be the best predictor of CV risk for all subsets of populations. LDL-C in South Asians is, surprisingly, often normal or lower,^10^, ^11^, ^12^ compared with their White Caucasian counterparts (Table 1). However, there is evidence that it is qualitatively abnormal, with a higher prevalence of more atherogenic small, dense particles – therefore, at any given LDL-C level, the risk of MI is higher for South Asians compared with other ethnicities.^11^, ^12^ Consequently, risk prediction using LDL-C alone may underestimate CV risk in South Asians.

**Table 1:** Dyslipidaemia among South Asians compared with White Caucasians.^13^, ^11^

Lipid variable	South Asians vs. White Caucasians
Total cholesterol	Similar or lower
LDL cholesterol	Similar or lower
HDL cholesterol	Lower
Triglycerides	Higher
Non-HDL cholesterol	Similar
Lipoprotein (a)	Higher
Small dense HDL	Higher
Small dense LDL	Similar
Total cholesterol/HDL ratio	Higher
Triglyceride/HDL ratio	Higher
Apo B	Higher
Apo B/Apo A ratio	Higher

Abbreviations: Apo A, apolipoprotein A; Apo B, apolipoprotein B; HDL, high-density lipoprotein; LDL, low-density lipoprotein.

### High-density lipoprotein cholesterol (HDL-C)

HDL-C is largely considered cardioprotective. South Asians consistently have lower HDL-C levels and more dysfunctional HDL particles, reducing CAD protection.^12^ However, HDL-C levels are not static; physical activity increases HDL-C, and improved glycaemic control improves HDL-C in those with diabetes.^9^, ^11^, ^14^, ^15^ These findings highlight the complex interplay of genetic and environmental factors.

### Apolipoproteins

Apolipoproteins (Apo) are important in maintaining the structural integrity and solubility of lipoproteins. Apo B levels correlate well with non-HDL cholesterol levels, while Apo A1 correlates with HDL-C levels.^16^ Evidence suggests that Apo B may be a more accurate predictor of CV risk than LDL-C. The INTERHEART study found that Apo B levels better predicted acute MI across populations, including South Asians, who exhibited elevated Apo B levels and an increased Apo B_100_/Apo A1 ratio, both linked to CAD risk.^8^, ^11^

Apo B may become a first-line marker for dyslipidaemia, though further research is needed to confirm its superiority over LDL-C. Meta-analyses support its use, and the European Society of Cardiology recognises it as an alternative to LDL-C measurement, particularly when triglycerides exceed 2 mmol/L, which can underestimate LDL-C.^17^ Given the high prevalence of hypertriglyceridaemia in South Asians, Apo B measurement should be considered, though challenges remain regarding accessibility, usability and its integration into risk scores.

### Lipoprotein (a) [LP(a)]

Lp(a) is structurally similar to LDL-C. High levels are independently associated with prematurity, severity and extent of coronary atherosclerosis.^18^ Extremely high Lp(a) levels of over 430 nmol/L convey a lifetime CVD risk similar to those with heterozygous familial hypercholesterolaemia (FH).^11^, ^18^

Lp(a) is highly heritable – approximately 90% of a person’s Lp(a) level is genetic. South Asian populations have higher Lp(a) levels than their White Caucasian counterparts; it is thought that Lp(a) is a significant contributor to residual CV risk in South Asians.^18^ Of note, South Asian immigrants have Lp(a) levels that are similar to their counterparts in their country of origin.^13^

At present, there is growing interest in Lp(a) lowering therapies and their possible CV benefit, both through current licensed LDL-C lowering therapies, and targeted novel agents. If proven effective, the benefit of such therapies could theoretically be greater in South Asians, given their high Lp(a) levels.

### Triglycerides

Evidence suggests that hypertriglyceridaemia confers a modest increase in CV mortality,^19^ independent of LDL-C level. Individuals of South Asian ancestry can exhibit significant hypertriglyceridaemia compared with their White Caucasian counterparts, with high triglyceride levels being twice as common in South Asians.^8^, ^10^, ^11^ As mentioned previously, triglyceride levels of over 2 mmol/L can lead to an underestimation of LDL-C, and so caution is required when interpreting LDL-C results in the context of hypertriglyceridaemia, in order to avoid underestimating CV risk.

## Cardiovascular risk assessment and diagnosis

To effectively identify those needing primary prevention, CV risk assessment strategies must be tailored to South Asians. In the UK, NICE [NG238] guidelines recommend the QRISK3 score. Lipid modification therapy is recommended in people aged 25–84 years if their 10-year risk of developing CVD is 10% or more, and lifestyle modification is ineffective or inappropriate.^19^

QRISK3, extensively validated, includes nine ethnic subcategories and applies a 1.3–1.7 risk multiplier based on gender and country of origin. However, with <5% South Asian participants in its derivation and validation cohorts, QRISK scores likely underestimate risk, particularly in South Asian women.^4^, ^20^, ^21^ Additionally, broad ethnic classifications (eg ʻIndian’ or ʻOther Asian’) fail to reflect population diversity. The recently published QRISK4 has included nine new risk factors, but is also limited by its genetically homogenous derivation and validation cohorts.^22^ Similarly, the USA uses the Pooled Cohort Equations, which also underestimates CV risk in South Asians.^4^ However, some risk scores do not incorporate ethnicity data, for example, ESC’s SCORE2 advises a crude multiplier of 1.4 for South Asians.^23^

While risk scores are effective at a population level, individual risk prediction requires careful evaluation. Emerging lifetime risk calculators may enable earlier primary prevention, but the QRISK3 lifetime model has also been shown to underestimate risk.^24^

These knowledge gaps underscore the need for large, representative, prospective trials with significant South Asian participation to improve CV risk prediction. However, other approaches to improving our assessment of CV risk in South Asians, such as measurement of Lp(a) or Apo B to identify those likely at higher risk, as well as emerging tools such as polygenic risk scores, must also be considered.^4^

## Familial hypercholesterolaemia (FH) in South Asians

Monogenic FH affects approximately 1 in 250–500 people in the UK,^25^, ^26^ with a similar prevalence being detected in South Asian populations^27^ However, detection is poor – over 85% of people with FH in the UK are unaware that they have the condition. Untreated, people aged 20–39 with FH have a 100-fold increased risk of death from CAD compared to those without FH, therefore early identification and treatment are crucial.^26^ QRISK scores are not validated for this population, and severely underestimate their risk.^28^ All patients with suspected FH should be referred to their local FH services for investigation and treatment.

At present, there is evidence of diagnostic inequality, with lower levels of diagnosis in non-native English speakers, Indian ethnic groups, and in areas of greater deprivation.^25^ Targeted screening of high-risk populations may increase identification.

Despite the higher risk of dyslipidaemia in South Asians, research on pathogenic FH variants in this group is limited. One study in India revealed variants differing from those identified in Western populations.^27^ Sequencing whole genes rather than testing for specific mutations may allow new mutations to be identified in less-studied populations.

## Management of dyslipidaemia

At present, LDL-C is the principal target in dyslipidaemia management. UK and European guidelines both recommend LDL-C targets based on calculated CV risk, and whether primary prevention or secondary prevention is needed, but they differ in specific thresholds, in part due to UK guidance incorporating cost efficacy into treatment targets.^17^, ^19^ Nonetheless, both agree that, overall, lower LDL-C levels are better for reducing CV risk, with ESC guidelines recommending an LDL-C of under 1.4 mmol/L in those with very high CV risk.^17^, ^19^ Ultimately each person’s individual risk should be considered when deciding treatment targets.

### Lifestyle modifications

Lifestyle modifications to reduce CV mortality, including diet and exercise, are essential for all populations. Furthermore, some patients prefer non-pharmacological approaches which benefit overall wellbeing and have fewer side effects than medications.

Physical activity and dietary interventions lower both LDL-C and triglyceride levels.^29^ Beyond improving dyslipidaemia alone, lifestyle modifications have also been shown to improve blood pressure control and promote weight loss,^29^ helpful given that South Asians should aim for a lower BMI than White Caucasians to reduce obesity-related complications.^11^ However, culturally tailored interventions reflecting community norms should be considered. Barriers to behaviour change include gender roles, cultural priorities, health education gaps and misconceptions about exercise, alongside socioeconomic limitations.^15^ However, the approach cannot be ‘one size fits all’ – the recent results of the SAHELI study demonstrated that a culturally adapted, group lifestyle intervention was not more effective than written health education materials for CVD risk factor reduction, highlighting the complexity of factors at play.^30^

### Medical therapies

**Fig. 2:**
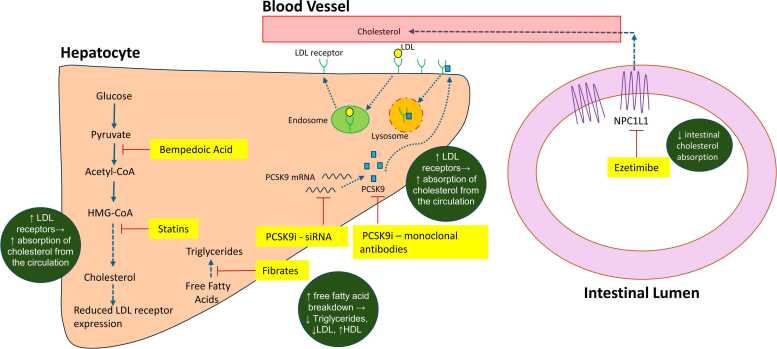
Lipid lowering therapies and their targets^8^, ^12^, ^17^, ^31^: Statins inhibit β-hydroxy β-methylglutaryl-CoA reductase; bempedoic acid works upstream to statins, inhibiting ATP citrate lyase; ezetimibe inhibits Niemann-Pick C1-like 1 (NPC1L1) protein, preventing intestinal cholesterol absorption; proprotein convertase subtilisin–kexin type 9 (PCSK9) can be blocked by PCSK9 inhibitors (PCSK9i), either directly by monoclonal antibody binding to PCSK9, or through small interfering RNA (siRNA) preventing PCSK9 messenger RNA (mRNA) translation; Fibrates are PPAR-alpha agonists and mediate changes in lipoprotein metabolism, leading to a reduction triglyceride and LDL synthesis.

We focus here on the medical management of non-familial hypercholesterolaemia (Fig. 2); patients with possible or definite FH, once commenced on a high-dose statin, should be referred to local FH services.

### Statins

Statins reduce cholesterol levels by being a selective competitive inhibitor of β*-*hydroxy β-methylglutaryl-CoA reductase, increasing hepatic uptake of LDL-C from the circulation. This has been extensively demonstrated to translate into a reduction in CV mortality.^5^ Statin therapy is the cornerstone of hypercholesterolaemia treatment, including in South Asians.^19^, ^32^

Statin-induced liver toxicity and myopathy occurs in 1–5% of patients.^33^, ^34^ While some research suggests that statins are well tolerated within the South Asian population,^35^ others indicate differences in metabolism, potentially increasing side effect risk. For example, rosuvastatin is only licenced up to a maximum dose of 20 mg in Asian patients due to a small-scale pharmacokinetics study of 36 Caucasian, 36 Chinese, 35 Malay and 35 Asian-Indian participants.^36^ In this study, there was a higher peak plasma concentration of statin within Asian participants, which could potentially increase the risk of statin-associated side effects when administered at higher doses – although this latter theory has yet to be demonstrated in practice. Additionally, the SLC01B1C allele, linked to statin-induced myopathy, was found in 15% of a Keralan cohort, suggesting possible genetic predisposition to myopathy in South Asians.^37^

Importantly, there does not appear to be a discrepancy in efficacy of statins between South Asian and White Caucasian populations.^34^ Atorvastatin and rosuvastatin are more effective than earlier statins, with some evidence suggesting greater LDL-C reduction with rosuvastatin in South Asians.^35^ For those experiencing muscular symptoms, exogenous vitamin D supplementation can improve statin-related muscular intolerance associated with low vitamin D, and therefore this insufficiency should be looked for and treated, particularly given its prevalence in South Asians.^38^ It is also important to note that the dose–response curve is non-linear, and the majority of statin’s LDL-C lowering abilities is seen at lower doses, with only small incremental reductions at higher doses. Therefore, maximally tolerated dosing is a viable strategy for those experiencing side effects.^35^ Moreover, evidence suggests that 90% of reported statin side effects are attributed to the nocebo effect,^39^ highlighting the importance of patient counselling and rechallenge strategies in those without biochemical evidence of toxicity.

### Ezetimibe

Ezetimibe inhibits intestinal cholesterol absorption. There is evidence for ezetimibe as combination therapy with statins in secondary prevention, including in South Asians.^40^ The INFINITY study found that in South Asian Canadians with CAD or diabetes and persistent hypercholesterolaemia, 76% of those on ezetimibe 10 mg OD plus statin achieved LDL-C <2.0 mmol/L in 12 weeks, compared with 48% with doubled statin doses. Ezetimibe was well tolerated.^40^ The IMPROVE-IT study, a large double-blind randomised controlled trial which included a small number of participants of South Asian descent, supports the results from the INFINITY study, with modest improvement in cardiovascular outcomes also seen.^41^

Combination drug therapy could be considered in all South Asian patients with uncontrolled dyslipidaemia at high risk of CVD; however, there is limited evidence for its use in primary prevention for all ethnicities.^19^

### Bempedoic acid

Bempedoic acid, an oral therapy inhibiting ATP-citrate lyase upstream of statins in hepatocytes, reduced LDL-C by 21% and major CV events by 13% in a randomised controlled trial of almost 14,000 patients. However, this was driven largely by rates of MI and revascularisation, with no reduction in mortality. It is an option for statin-intolerant or statin-contraindicated patients, particularly as it is not associated with myalgia or myopathy – unlike statins, bempedoic acid is activated in the liver, and not in skeletal muscle. The side effects may include gout, anaemia and constipation.^42^ Further research is needed on its efficacy in South Asians and in combination with ezetimibe.

### Fibrates

A raised triglyceride level is a risk factor for CVD, independent of total cholesterol. However, hypertriglyceridaemia is often secondary, and so treatment of the underlying cause, such as hypothyroidism and insulin resistance, is essential. When targeted treatment is required, fibrates (peroxisome proliferator receptor-ɑ agonists) are usually first line.^19^ Gastrointestinal side effects are common; rarely, myositis or rhabdomyolysis can occur.

To date, there are no studies looking at the efficacy of fibrates within the South Asian population.^9^ While the FIELD and ACCORD trials did not demonstrate any improvement in CV mortality, subgroup analysis of both identified a specific beneficial role in treating men with high triglycerides (>2.3 mmol/L) and low HDL-C (<0.9 mmol/L) after statin therapy has reduced their LDL-C levels.^43^, ^44^ Given the dyslipidaemia patterns in South Asian populations, there is a theoretical benefit of fibrates in South Asians with triglyceridaemia despite statin therapy, which should be explored.

### Icosapent ethyl

Icosapent ethyl, a high-dose omega-3 fatty acid, is recommended by NICE for use as an adjunct to statins for persistent hypertriglyceridaemia (>1.7 mmol/L).^45^ While the data remain inconclusive, the REDUCE-IT trial demonstrated a reduction in triglyceride levels and a 25% relative risk reduction in major adverse cardiac events, as compared to the mineral oil placebo group. However, it is noted that the placebo group had a 10% increase in LDL levels, weakening this conclusion. The mechanism of icosapent ethyl is unclear, and non-lipid effects may contribute to any clinical efficacy.^17^ Further ethnicity-specific data are required to see whether it is of benefit to South Asians with persistent hypertriglyceridaemia.

### Proprotein convertase subtilisin/kexin type 9 (PCSK9) inhibitors

PCSK9 regulates the degradation of the LDL receptor, therefore its inhibition enhances cholesterol uptake into hepatocytes, effectively lowering LDL-C. It also reduces Lp(a), an effect that is being further investigated.^31^ At present, we have two methods of PCSK9 inhibition in humans – monoclonal antibodies or small interfering RNA (siRNA). Both require injection, with monoclonal antibodies (alirocumab and evolocumab) administered fortnightly, while siRNA inhibitors (inclisiran) silence PCSK9 mRNA, enabling six-monthly dosing. Large-scale randomised control trials studying alirocumab or evolocumab alongside statins, with at least 2.2 years of follow-up, demonstrated a 15% relative risk reduction in major adverse cardiac events compared with placebo.^46^, ^47^ Initial evidence suggests that outcomes across ethnicity are similar. For example, the Fourier trial of evolocumab included 13% of patients of Asian origin, and no difference in LDL-C reduction between ethnicities was found, although this was underpowered to look at this.^47^, ^48^ Pooled analysis of ORION trials found that inclisiran provided sustained 50% LDL-C reductions across diverse patient groups, with only mild adverse effects over 4 years, though long-term outcome data are awaited.^49^

However, these therapies are costly, and incorporation into guidelines are regulated by NICE in the UK. NICE currently recommends monoclonal antibodies for patients without FH based on cardiovascular risk, with an LDL-C threshold of ≥3.5 mmol/L; Inclisiran is recommended for those with LDL-C ≥2.6 mmol/L.^19^ The aforementioned trial data highlight the potential benefits of PCSK9 inhibitor combination therapy for high-risk South Asians who fail to achieve LDL-C targets on conventional therapy. Additionally, inclisiran may be particularly beneficial for patients with poor adherence, as its twice-yearly dosing regimen offers a promising approach to lipid control.

## Improving therapy uptake and adherence

Non-adherence to lipid-lowering therapies is linked to adverse outcomes, with evidence suggesting that South Asians with CVD are less likely to adhere than White Caucasians.^50^

A narrative review identified multifaceted reasons for non-adherence in South Asians, including medication side effects, cost, forgetfulness, dosing frequency, illness stigma and language barriers.^50^ Additionally, patients do not perceive symptoms or immediate benefits from statins, making adherence less likely. Others attribute CAD to fate or prefer traditional remedies over prescribed treatments.^51^

Factors associated with improving adherence include increased knowledge regarding the medications,^50^ memory mechanisms and pre-packaging of medications,^52^ and a rosuvastatin–ezetimibe combination pill (thereby reducing pill burden), though the latter is yet to be trialled in a South Asian population.^53^

Research on injectable therapies like PCSK9 inhibitors in South Asians is limited, but studies on insulin use highlight barriers such as fear, social stigma, inconvenience and lack of education.^51^ However, not all issues found with injectable medications may be applicable to PCSK9 inhibitors, due to its less frequent dosing.

Interventions improving adherence require tailored patient education and support, and therefore necessitate a multifaceted approach that fosters patient and clinician engagement.

## Conclusion

South Asians have more pro-atherogenic lipid profiles and higher CV risk, warranting a personalised approach to dyslipidaemia. Worldwide, South Asian ancestry accounts 23% of the global population. With growing healthcare needs of an ageing population with multiple long-term conditions, CVD prevention is a research and policy priority. Timely identification and treatment of hyperlipidaemia are key to reducing CV morbidity and mortality. Patient and clinician education and engagement is key.

## Key practice implications

• South Asians are more likely to have pro-atherogenic lipid profiles, and risk prediction using LDL-C alone may underestimate CV risk in South Asians, due to more dysfunctional lipoproteins which confer a higher risk of CVD at lower LDL-C levels.
• Triglyceride levels of over 2 mmol/L can lead to an underestimation of LDL-C, and so caution is required when interpreting LDL-C results in the context of hypertriglyceridaemia.
• Apo-B measurement should be considered, given the high prevalence of hypertriglyceridaemia in South Asians, though challenges remain regarding accessibility, usability, and its integration into risk scores.
• Current risk scores may underestimate CV risk in South Asians.
• Detection of familial hypercholesterolaemia (FH) remains low, and all those with suspected FH should be referred to local FH services without delay.
• There does not appear to be a discrepancy in efficacy of statins between South Asian and Caucasian populations, and this remains first-line medical therapy. Other treatments should also be considered, including injectable therapies.
• Improving adherence and understanding requires a multifaceted approach that includes patient education and culturally tailored approaches.

## CRediT authorship contribution statement

**Lavandan Jegatheeswaran:** Writing – original draft. **Haneesh Kaur Johal:** Writing – original draft. **Aneesha Chauhan:** Writing – review & editing, Writing – original draft, Visualization, Project administration, Conceptualization. **Shaan Sahota:** Writing – original draft, Visualization. **Prashanth Patel:** Writing – review & editing. **Pankaj Gupta:** Writing – review & editing. **Rubin Minhas:** Writing – review & editing. **Kiran Patel:** Writing – review & editing, Supervision. **Anvesha Singh:** Writing – review & editing, Supervision. **Vinod Patel:** Writing – review & editing. **Amitava Banerjee:** Writing – review & editing.

## Funding

This research received no specific grant from any funding agency in the public, commercial or not-for-profit sectors. Other acknowledgements: Pankaj Gupta (PG) is funded by the National Institute for Health and Care Research (NIHR) Leicester Biomedical Research Centre (BRC). The views expressed are those of the author(s) and not necessarily those of the NIHR or the Department of Health and Social Care. PG has received unrestricted grants for audits/quality improvement projects, attendance of conferences and speaker fees from Amgen and Sanofi and a grant for attendance of a conference from Daiichi Sankyo.

## Declaration of Competing Interest

The authors declare that they have no known competing financial interests or personal relationships that could have appeared to influence the work reported in this paper.
